# 1336. Presence of Antibodies to non-SARS-CoV-2 Associates With Generation of Neutralizing Antibodies to SARS-CoV-2 Following mRNA Vaccination

**DOI:** 10.1093/ofid/ofad500.1173

**Published:** 2023-11-27

**Authors:** Roukaya Al Hammoud, Gabriela P Del Bianco, Anoma Somasunderam, Stacy Gomez Hernandez, Kaleigh Riggs, Syed Hashmi, James Murphy, Gloria Heresi

**Affiliations:** UT Houston- McGovern Medical School, Houston, Texas; UTHealth, McGovern Medical School, Houston, Texas; UTHealth, McGovern Medical School, Houston, Texas; UTHealth, McGovern Medical School, Houston, Texas; University of Texas Health Science Center, McGovern Medical School, Houston, Texas; University of Texas Health Science Center, McGovern Medical School, Houston, Texas; UTHealth, McGovern Medical School, Houston, Texas; UTHealth, McGovern Medical School, Houston, Texas

## Abstract

**Background:**

The immunological interplay between SARS-CoV-2 immunization, SARS-CoV-2 infection, and exposure to other human coronaviruses is poorly understood. Therefore, we evaluated the relationship of the humoral immune responses to SARS-CoV-2 and selected other human coronaviruses (HCoVs).

**Methods:**

We enrolled 42 healthcare workers (HCWs) in a Pediatric Department at a medical school in Houston, Texas, during a Delta variant dominance period (2021) of the SARS-CoV-2 pandemic. All had two immunizations with mRNA SARS-CoV-2 vaccines at a median of 7.4 months before sample collection. Using multiplex assays, we tested binding antibodies (Ab) and neutralizing antibodies (NAb) to SARS-CoV-2 and Ab responses to selected non-SARS-CoV-2 HCoVs.

**Results:**

100% had Ab to all components of a SARS-CoV-2 screening panel that comprised receptor-binding protein (RBD), spike 1 (S1), and spike 2 (S2). 13 out of 42 (31%) had Ab to nucleocapsid antigen (N) suggesting previous coronavirus infection of whom 7 (53%) reported a previous PCR-confirmed SARS-CoV-2 infection and had significantly higher Ab response to each of the four screening antigens. (Table 1) Although all volunteers had notable levels of SARS-CoV-2 specific Abs, only 42% had NAb to SARS-CoV-2. Notable findings for volunteers with NAb included a high frequency of NAb to Wild Type (100%), Alpha (72%) and Delta variants (72%). More mRNA-1273 (Moderna) recipients (61%) had NAb than BTN162b2 (Pfizer) (25%) (*p*-value: 0.041).

83% of volunteers had detectable Ab to at least one of HCoVs. Ab to SARS-CoV-1 is strongly associated with a positive NAb response to SARS-CoV-2 (44% versus 0, p-value 0.001).

**Table 1**

**Figure:**
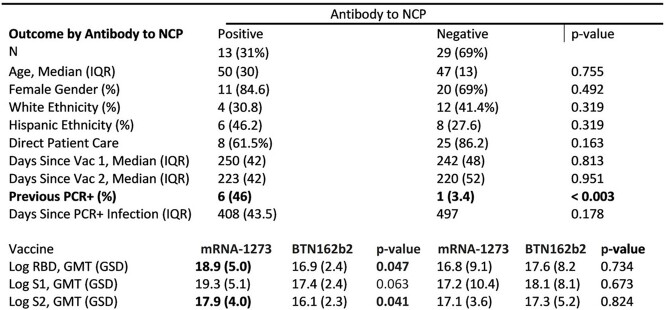

Demographic and serological profile

**Table 2**

**Figure:**
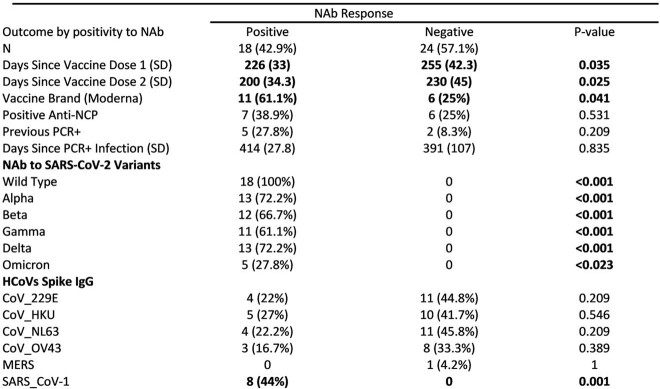

Comparison between participants with Neutralizing Ab response and those without

**Conclusion:**

Generation of SARS-CoV-2 NAb following mRNA vaccination of HCWs is strongly associated with the presence of Ab to SARS-CoV-1. The source of SARS-CoV-1 Ab in our population is unknown. The nearly 20% prevalence of SARS-CoV-1 Ab exceeds the known prevalence of SARS-CoV-1 infection. There is may be a neutralization relevant shared immunogen between SARS-CoV-1 and SARS-CoV-2.

**Disclosures:**

**All Authors**: No reported disclosures

